# The Incorporation of Zinc into Hydroxyapatite and Its Influence on the Cellular Response to Biomaterials: A Systematic Review

**DOI:** 10.3390/jfb15070178

**Published:** 2024-06-28

**Authors:** Jessica Dornelas, Giselle Dornelas, Alexandre Rossi, Adriano Piattelli, Natalia Di Pietro, Tea Romasco, Carlos Fernando Mourão, Gutemberg Gomes Alves

**Affiliations:** 1 NanoOnco3D, Rio de Janeiro 20000-000, Brazil; 2 Cell and Molecular Biology Department, Institute of Biology, Fluminense Federal University, Niteroi 24220-900, Brazil; 3 Post-Graduation Program in Sciences & Biotechnology, Institute of Biology, Fluminense Federal University, Niteroi 24220-900, Brazil; 4 CBPF–Brazilian Center for Research in Physics, Rio de Janeiro 22290-180, Brazil; 5 School of Dentistry, Saint Camillus International, University of Health and Medical Sciences, 00131 Rome, Italy; 6 Department of Medical, Oral and Biotechnological Sciences, Center for Advanced Studies and Technology-CAST, “G. D’Annunzio” University of Chieti-Pescara, 66100 Chieti, Italy; 7 Department of Periodontology, Tufts University School of Dental Medicine, Boston, MA 02111, USA

**Keywords:** zinc, hydroxyapatite, ZnHA, absorbable implants, osteoblasts, dental biomaterials, regenerative dentistry

## Abstract

Zinc is known for its role in enhancing bone metabolism, cell proliferation, and tissue regeneration. Several studies proposed the incorporation of zinc into hydroxyapatite (HA) to produce biomaterials (ZnHA) that stimulate and accelerate bone healing. This systematic review aimed to understand the physicochemical characteristics of zinc-doped HA-based biomaterials and the evidence of their biological effects on osteoblastic cells. A comprehensive literature search was conducted from 2022 to 2024, covering all years of publications, in three databases (Web of Science, PUBMED, Scopus), retrieving 609 entries, with 36 articles included in the analysis according to the selection criteria. The selected studies provided data on the material’s physicochemical properties, the methods of zinc incorporation, and the biological effects of ZnHA on bone cells. The production of ZnHA typically involves the wet chemical synthesis of HA and ZnHA precursors, followed by deposition on substrates using processes such as liquid precursor plasma spraying (LPPS). Characterization techniques confirmed the successful incorporation of zinc into the HA lattice. The findings indicated that zinc incorporation into HA at low concentrations is non-cytotoxic and beneficial for bone cells. ZnHA was found to stimulate cell proliferation, adhesion, and the production of osteogenic factors, thereby promoting in vitro mineralization. However, the optimal zinc concentration for the desired effects varied across studies, making it challenging to establish a standardized concentration. ZnHA materials are biocompatible and enhance osteoblast proliferation and differentiation. However, the mechanisms of zinc release and the ideal concentrations for optimal tissue regeneration require further investigation. Standardizing these parameters is essential for the effective clinical application of ZnHA.

## 1. Introduction

In implant dentistry, chronic diseases or accidental damage to bone tissue can significantly impact patients’ quality of life, leading to extensive dental treatments, increased healthcare costs, and economic burdens [1]. Effective bone regeneration is crucial for the success of dental implants, which are often required due to tooth loss or severe periodontal diseases. Possible treatments with exogenous and autogenous grafts are employed to accelerate the regeneration of injured tissue [2]. However, even as a gold standard, exogenous grafts have limitations of use due to severe infections and rejections, whereas autogenous ones have limitations in donor tissue availability and complications related to a second surgery site for its removal [3]. In this context, alloplastic biomaterials still represent an alternative to achieving tissue repair because they are biocompatible and osteoconductive, enabling accelerated tissue regeneration and increased patient quality of life [4].

Several alloplastic materials can be used for bone repair, including metallic grafts, polymers, ceramics, and calcium phosphates (CaP). Hydroxyapatite (Ca_10_(PO_4_)_6_(OH)_2_ or HA) is the CaP most similar to the inorganic portion of bone tissue [5]. However, synthetic HA has high crystallinity and low resorption, making implant osteointegration difficult and increasing tissue regeneration time [6]. On the other hand, using ionic substitutions in the HA structure may enhance the material physicochemical properties, such as reduced crystallinity, increasing solubility [7]. Furthermore, bioresorbable materials may release biologically active incorporated ions, such as Sr^2+^, Mg^2+^, CO_2_^3−^, and Zn^2+^. The release of these ions from a designed “smart material” is expected to stimulate important biological responses [8].

Even as a trace element in the body, zinc is vital for metalloproteinase enzyme activity and increases the expression of osteoblast differentiation-linked proteins such as the RUNX family transcription factor 2 (RUNX2), alkaline phosphatase (ALP), osteocalcin (OCN), and type I collagen (COL1), thus having an important role in bone metabolism [9,10]. Anti-inflammatory effects and antibacterial activity are also reported for this ion [11], while its deficiency is the genesis of various oromaxillary diseases [12]. Several studies investigated HA-based biomaterials incorporated or functionalized with zinc, identifying important biological responses in vitro and in vivo [13]. Developing zinc-doped biomaterials is often based on the beneficial effect of accelerating tissue regeneration, ensuring biological properties such as biocompatibility, osseointegration, and osteoconductivity [9]. However, in vivo studies on this theme remain controversial. While some studies reported no improvement in bone formation by the incorporation of zinc into HA [14], others indicate a good performance of hydroxyapatite substituted by zinc (ZnHA) in the filling of bone defects [15]. Furthermore, the impact of different zinc concentrations and the methods of substitution/incorporation for promoting bone regeneration remains unclear [13].

In this context, in vitro studies performed with osteoblasts, the main bone cells involved in bone regeneration, may provide evidence in controlled environments to understand the bone response to zinc-doped HA at a cellular level, and the mechanisms of action involved in ZnHA-mediated bone regeneration.

Therefore, the purpose of this systematic review was to assess the scientific literature and qualitatively evaluate the available in vitro evidence on the response of osteoblasts to biomaterials based on ZnHA, and the identified molecular mechanisms involved in bone tissue regeneration, providing insights for the development of advanced biomaterials for dental implants and regenerative medicine.

## 2. Materials and Methods

This systematic review was conducted according to the protocol registered at the Open Science Framework database, available at https://doi.org/10.17605/OSF.IO/948YC, and reported according to the PRISMA Statement (Supplementary Checklist) [16]. An electronic search was conducted from March 2022 to 9 May 2024, in three different databases: PubMed (MEDLINE), Scopus (Elsevier), and Web of Science (WoS, Claryvate Analytics). The search on PubMed was conducted with the search key (osteoblast* OR bone OR bone cell* OR MSC OR “mesenchymal stem cell”) AND (hydroxyapatite OR HA) AND (zinc (tiab) OR Zn (tiab)) AND (in vitro). The same search key was adapted for the other databases according to their syntax rules, without limits or filters applied, including no limits at the time of publishing. Google Scholar was also consulted as a source of grey literature, limited to complete studies with original results.

### 2.1. Eligibility Criteria

Eligibility criteria included articles in any language, following the PECOS modified criteria:

- P (population) = osteoblastic or bone cells;
- E (exposition) = direct or indirect exposure to calcium phosphates doped with zinc;
- C (comparison) = calcium phosphates not containing zinc;
- O (outcome) = biocompatibility, proliferation, differentiation, mineralization, and gene expression;
- S (setting) = in vitro tests.

The exclusion criteria applied to entries characterized as review articles, patent applications, book chapters, theses, articles using a mixture of ions other than zinc, performing only physicochemical, antibacterial, in vivo assessments, or assessing cell models not related to osteoblasts or mesenchymal stem cells. Some articles were considered off-topic because they did not relate to any of the topics researched; for example, non-ceramic biomaterials or pharmaceutical studies of zinc-containing drugs.

### 2.2. Selection of Articles

Initially, the titles and abstracts of the articles were analyzed, and those meeting the eligibility criteria were selected. Using tools from the Mendeley program (Elsevier Ltd., London, UK), duplicate articles with the same title and abstract were excluded. The remaining articles were fully read and analyzed according to the eligibility criteria. The screening was performed by two authors previously calibrated. Disagreements about the article’s eligibility were resolved by discussing the article’s relevance with a third author.

### 2.3. Quality Assessment of the Selected Studies

All selected articles were evaluated using the Toxicological Data Reliability Advisory Tool (ToxRTool, Brussels, Belgium), a standardized guide to the inherent quality of toxicity data. Eighteen criteria were considered, which describe fundamental points for the study, such as identification of test substance, test system, study design, and results analysis. Articles that totaled less than 11 points were characterized as unreliable, between 11 and 14 are reliable but with restrictions, and articles with scores above 15 points are considered reliable without restriction. Two previously calibrated authors performed the evaluation, with disagreements solved by discussions with a third author.

### 2.4. Data Extraction and Qualitative Synthesis

Data extraction was divided into two steps. The first considered the characteristics of the material used in the study, such as the material type, amount of zinc released in solution, and how much was used in titanium coating and biocomposites. The second stage evaluated the result of modifying the biomaterial’s extract and/or direct contact in a biological system. In this sense, data regarding the cell type used in the studies, type of test performed, and main obtained results compared to an experimental control were collected, tabulated in an Excel spreadsheet, and qualitatively evaluated for the main similarities and differences.

## 3. Results

### 3.1. Database Search

**Figure 1:**
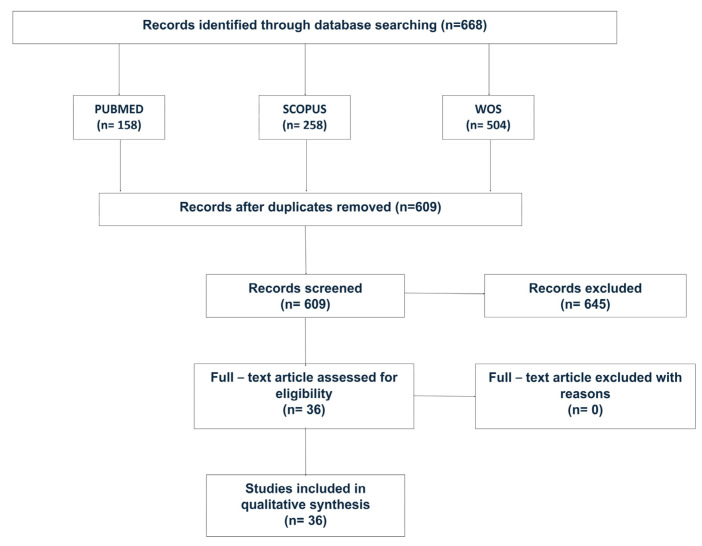
PRISMA flowchart for the systematic review detailing the database searches, the number of abstracts screened, and the full texts selected for the analysis.

Using the search key (Figure 1), the entries were obtained in three databases, PubMed, Scopus, and WoS. Initially, 158 articles were selected from PubMed, 258 from Scopus, and 504 from WoS database, totaling 920 entries.

A total of 311 duplicate articles were excluded. Using the selection criteria, the remaining 609 articles were evaluated. Table 1 shows the number of excluded articles related to the evaluated criteria. Then, 36 articles were evaluated in detail and used to determine the results found in this review.

### 3.2. Quality Assessment

All selected articles passed the quality assessment and presented a good design, and they are all classified as reliable, even though eight articles presented some restrictions. Articles that quantified the concentration of zinc released and incorporated into the biomaterial highlighted the importance of zinc quantification on the biological effect (Table 2).

### 3.3. Characteristics of Selected Studies

The selected studies used different HA-doped zinc concentrations incorporated in different formulations of HA materials, including coatings, nanocomposites, nanoparticles, discs, powders, and granules, as shown in Table 3. Some studies evaluated the release of zinc in an aqueous medium to determine the dissolution potential.

Several studies reported changes in HA’s crystalline structure after zinc incorporation. The methods of analysis used by the authors are described in Table 3. X-ray diffraction (XRD) was the main technique used to study alterations of HA crystallinity after introducing Zn^2+^ (12 articles). As a complementary technique, the Rietveld powder structure refinement method was used to detail the characteristics of the crystal structure after replacements [56]. Other techniques, such as scanning electron microscope (SEM), selected area electron diffraction (SAED), and transmission electron microscope (TEM), were used; however, they do not guarantee the same comparison with standard materials [37,46,55].

Most authors performing XRD assessments identified crystalline changes from the incorporation of Zn^2+^. They observed the contraction in the crystal lattice, which promoted deformities in the crystallite, favoring solubility and altering the surface parameters of the materials (Table 4).

Other parameters, such as chemical composition and degradation of biomaterial in aqueous solvents, were evaluated based on these changes. Many biological tests have been used to validate the effects of zinc doping on HA. Cytotoxicity was assessed using osteoblasts, pre-osteoblasts adipose, and mesenchymal stem cells from both human and animal origin, and mainly employing the 3-(4,5-dimethylthiazol-2-yl)-2,5 diphenyl tetrazolium bromide (MTT) test. Using this method, zinc at low concentrations showed no cytotoxic effect. Cell proliferation was also assessed in several studies, with an overall stimulation of osteoblast growth during exposure to zinc-containing biomaterials. However, only a few studies have investigated the effect of doped materials on the production of osteogenic proteins. Among those, ALP activity and its gene expression were the most represented, and other molecular factors, such as COL1, OCN, RUNX2, Osterix, and bone morphogenic protein 2 (BMP2), were also analyzed (Table 5).

## 4. Discussion

### 4.1. The Incorporation of Zinc into Hydroxyapatite

Although synthetic HA is similar to the organic composition of bone tissue, natural HA presents many different ions incorporated [57]. Zinc is one of the trace elements found in the bone matrix that, when incorporated into the HA structure, causes changes in its crystalline structure, modifying crystallinity, network organization, solubility, surface conditions, and ion leakage, with possible impact on the metabolism and behavior of osteoblasts that may improve osteoconduction and osteointegration [58].

In the present review, 25 studies incorporated Zn^2+^ into the HA structure with such intention. Most of them identified crystalline changes from the Zn^2+^ incorporation into the biomaterial (Table 3), allowing Zn^2+^ release in the periphery of the implant. The most common alteration on the crystalline structure occurs due to the Ca^2+^ substitution, promoting the contraction of the crystalline lattice, because Zn^2+^ presents a smaller atomic radius (134 pm) compared to Ca^2+^ (180 pm), resulting in the reduction of the ZnHA unit cell size [14]. According to Lala et al. [56], the increase in crystallinity is intensified with variations in the concentration of Zn^2+^ incorporated in the HA structure since the deformity of Ca^2+^ removal changes the crystallite plane c (height), thus reducing the unit cell crystallinity. In contrast, the ab (base) plane remains almost unaltered. The saturation of Zn^2+^ substitution is attained when its concentration reaches 15 mol%. In contrast, despite using the XRD technique, other studies did not find significant changes in material crystallinity [33,34,35,49,55]. Bhowmick et al. [20] did not observe changes in the crystal structure of HA after Zn incorporation. This is due to the presence of ZnO-shaped zinc, as Thian et al. [46] showed low incorporation of Zn^2+^ (1.6%) in the structure of HA.

This deformity in HA crystal structure alters other material parameters such as chemical composition, surface area, pore size, and pore volume [59]. As previously mentioned, stoichiometric synthetic HA shows Ca, P, and O at well-determined concentrations, giving a Ca/P ratio = 1.67. ZnHA is formed by replacing part of Ca^2+^ with Zn^2+^, which delays the nucleation of HA and renders the material calcium-deficient, as can be observed through the altered Ca/P ratio. Several of the identified studies from this review have described the assessment of the actual incorporated Zn2+ concentrations (Table 3). During the synthesis of the material, while theoretical Zn^2+^ concentrations are set from the calculation of reagent concentrations, the actual incorporation of Zn^2+^ in the samples tends to be lower than initially determined due to competition between Zn^2+^ and Ca^2+^ during nucleation. By quantifying the concentration of Zn^2+^ incorporated into HA, it is possible to decide on the actual concentration of Zn^2+^ included in the crystal network and monitor the release of this ion when in solution.

The main techniques used to identify the chemical composition of the elements in the selected articles were energy-dispersive X-ray spectroscopy (EDX), X-ray photoelectron spectroscopy (XPS), inductively coupled plasma optical emission spectroscopy (ICP-OES), atomic absorption spectroscopy (AAS), and X-ray fluorescence (XRF). The EDX and XPS techniques mainly analyze the surface chemical composition, which is ideal for evaluating ZnHA-coated materials but inefficient for total bulk quantification. In contrast, the ICP, XRF, and AAS techniques allow for quantifying ions present throughout the bulk of the material. As shown in Table 2, the concentrations of Zn^2+^ incorporated in the different materials ranged from 0.0009% to 2.9%. This information allows us to estimate and investigate the gradual release of Zn^2+^ in the implant and identify the dose/effect relationship of the zinc concentrations with the observed biological changes [41].

### 4.2. Solubility and the Release of Zn^2+^

According to the principles of regenerative medicine, to promote bone healing, a biomaterial should be simultaneously replaced by new bone tissue [15]. However, HA usually presents low solubility due to its high crystallinity. Ionic incorporations have been proposed as alternatives to solve this problem by inducing disorganization of the HA crystal lattice and making the material more soluble [60]. Considering bioactive innovative materials, substituting biologically active ions such as Zn^2+^ could theoretically combine the improved solubility with the induction of desired biological responses in bone metabolism, thus accelerating tissue regeneration [61]. Therefore, zinc-doped HA’s solubilization and release dynamics are essential factors to consider during in vitro and in vivo studies. However, only five selected studies verified the changes in Zn^2+^ content of the conditioned media before or during cell exposure. Several solutions were used for solubility tests, such as simulated body fluid (SBF), culture medium, Tris-HCl Buffer, and phosphate buffer-saline (PBS) [28,33,35,41,54]. De Lima et al. [35] showed that the ZnHA containing 1% Zn^2+^ released 1 ppm of this ion in cell culture media, modifying the ionic composition of the medium. This concentration is safe for cell viability, with no apoptotic effect on osteoblasts. Due to the different compositions of the studied solutions, it is difficult to identify whether the release of Zn^2+^ in various aqueous solutions will behave similarly. Gustavsson et al. [7] investigated the interaction of calcium phosphates in two media (DMEM and McCoy). They noted that the adsorption and desorption of Ca^2+^ and Pi ions are altered depending on the chemical composition of the aqueous medium and the exposure time.

### 4.3. Cell Adhesion into ZnHA Surfaces

Considering the difficulty of standardizing in vitro assays complementing these results, the systematic review by Cruz et al. [13] has shown controversy among authors considering a zinc-doped CaP (ZnCaP) bioreaction. Most studies have observed low resorption, but some authors have observed significant resorption in ZnCaP. This variation is related to the various implant types, shapes, sizes, and chemical composition, which can completely change the material structure and function, as observed in the present study (Table 2). It is essential to consider that the in vivo resorption process depends on material composition, biological fluid action for passive degradation, and osteoclast activity [62]. Animal model and implantation site variations may alter results as different cell types respond differently to biomaterials (Table 4).

Surface structures of biomaterials at the micro/nanoscale are often intentionally altered to modulate cellular behavior in parameters such as adhesion, morphology, differentiation, and migration. Modifications such as variation in roughness, porosity, cluster formation, and protein adsorption affect the biological response of osteoblastic cells [63]. These parameters directly influence the biological behavior of osteoblastic cells, promoting an adequate surface for cell adhesion that is intimately connected to the material osteoconductive properties necessary for graft integration to bone. The study from He et al. [28] observed that the more significant deposition of ZnHA on the substrate surface increases its roughness, which promotes more excellent cell adhesion. However, the biological effects of Zn^2+^ related to changes in porosity are controversial. Some studies reported more excellent cell adhesion in more porous samples [18], and the production of ZnHA aggregates with different concentrations of zinc increased cell adhesion [37,49,55]. In contrast, Li et al. [33,34] and De Lima et al. [35], while evaluating 1% ZnHA, observed more excellent cell adhesion without changes in porosity, attributing this to the biological effects of the released zinc in the culture media. Other studies found higher cell adhesion on materials with smaller grain sizes [24,51]. In contrast, incorporating Zn^2+^ into the HA structure did not change grain formation and, consequently, did not impact cell adhesion. Similarly, studies involving the adhesion of preosteoblasts onto 5% ZnHA nanostructured surfaces and MSCs seeded over macroporous chitosan–agarose scaffolds with Zn-doped nano-hydroxyapatite did not find effects for Zn^2+^ incorporation. In contrast, adhesion was significantly increased with doping with magnesium ions [32,47]. According to the authors, Mg^2+^ doping would be more efficient in supporting cell adhesion and spreading due to increased interactions with membrane-associated adhesion receptors like integrins [32].

In addition to surface properties playing an essential role in cell adhesion, studies showed that protein functionalization on the surface of materials improves cell adhesion. The main proteins and peptides tested were fetal bovine serum (FBS), RGD (Arg–Gly–Asp), albumin, laminin, denatured collagen, fibronectin, and vitronectin. Zhang [54] observed increased adhesion in ZnHA samples related to protein adsorption. These findings were corroborated by Ghorbania et al. [27], when testing FBS protein adsorption, finding a higher number of cells adhered to zinc-doped material, similar to Mavropoulos et al. [39] and Webster et al. [52]. Even with the incorporation of Zn^2+^ in the crystalline structure of HA, no change in the fundamental structure of the material was observed. Direct cell adhesion with doped-HA did not significantly improve the interaction of cells with the material. However, indirect factors such as Zn^2+^ release and surface protein adsorption contributed to cell adhesion. Surface modifications of ZnHA increase protein adsorption, which increases cell adhesion, a feature that can make the material more interactive with cells and make it more osteoconductive.

### 4.4. Effects on Cytocompatibility

Cytotoxicity assessment is a crucial first step in evaluating the effects of zinc-doped biomaterials, as it determines the biocompatibility and safety of the biomaterials intended for bone tissue engineering [64]. While Zn^2+^ is generally expected to promote cell proliferation and osteogenic differentiation, high concentrations of Zn^2+^ can potentially cause cell death. Controversies were found regarding the concentration of Zn^2+^ in doped HA that would maintain the material cytocompatibility. Lima et al. [35] observed the release of Zn^2+^ (0.8 ppm) from 1% ZnHA in conditioned media exposed to cells, relating the release of ions to increased cytocompatibility with primary human osteoblasts through a multiparametric method (XTT, Neutral Red, and CVDE). Bhowmick et al. [20] described that MG-63 cells in contact with ZnHA samples (5%, 10%, and 15%) showed a significant increase in cell viability compared to the undoped samples. On the other hand, cytotoxic effects were demonstrated by Luo et al. [37] and Li et al. [34], describing that high concentrations of Zn^2+^ (from 8 to 30%) may reduce cell proliferation and may be related to the cytotoxic effect of these concentrations. Similarly, Forte et al. [36] identified a significant reduction in osteoblast viability when indirectly exposed to 8% ZnHA. Still, this deleterious effect was counterposed by adding polyethyleneimine (PEI), producing a biocompatible, bi-functionalized material. More recently, Huang et al. [31] also identified cytotoxic effects of ZnHA in osteosarcoma cells (MG-63) but not in healthy MSCs and osteoblasts, suggesting an anticancer trait for this material. This ZnHA reduced the tumor size when implanted with a PCL scaffold on a murine in an in vivo model [31].

### 4.5. Effects on Cell Proliferation

Cell proliferation is the second step in establishing bone regeneration, supporting cell differentiation followed by forming new bone tissue, simultaneously with resorption of the biomaterial [65]. Most articles have confirmed the increase in cell density in HA with different concentrations of Zn^2+^ doping. Increased proliferation has been observed in several studies (Table 5). The presence of Zn^2+^ has been shown to benefit the proliferation of different cell types in the concentration range of 1% to 20%. Studies showed that ZnHA coverage on the titanium surface improved cell proliferation, and Zn^2+^ incorporation at concentrations from 0.0025 M to 0.56 M was promising [50,54]. From the ZnHA coating, Zn^2+^ release was observed in the 1% to 9% range [24,41], contributing to increased cell proliferation. Other materials, such as blocks, pellets, and biocomposites, have been shown to alter cell proliferation [18,53]. In contrast, polymers and collagen-associated materials seem to have masked the effect of doped biomaterial, not promoting proliferation [27,66]. The effect of Zn^2+^ concentration was dose-dependent, as shown by Zhong and Ma [55] and Wang et al. [50], as proliferation was increased in samples with a higher Zn^2+^ concentration (5% and 0.56 M, respectively). However, Okada et al. [42] have shown a dose-dependent reduction in murine pre-osteoblast proliferation when exposed to 15% ZnHA, suggesting once again harmful effects at higher Zn^2+^ incorporations.

### 4.6. Effects on Cell Differentiation and In Vitro Mineralization

Pre-osteoblast differentiation marks the beginning of new bone tissue formation [67]. Among the primary osteogenic markers are ALP, OCN, Col I, RUNX-2, Osterix, and BMP-2 [68]. Zn^2+^ plays a vital role in osteogenesis since this element’s absence delays the bone formation process [69]. Increased ALP activity is directly related to the ability of cells to activate signaling pathways favoring the formation of new bone tissue. Since this enzyme’s activity shows peaks of activity in the early periods of osteoblast cell differentiation, between the 14th and 21st day, varying according to cell line, most studies monitored the activity of this enzyme within this timeframe [22,28,29,30,37,38,44,49,50,51,53,55]. ALP activity was dose-dependent, as it gradually increased activity in samples with higher zinc concentration, according to Wang et al. [50]. ALP activity increased after exposure to materials doped with Zn^2+^ in the 5 to 20% concentration range. The studies by Zhong and Ma [55], Luo et al. [37], and Wang et al. [50], employing ZnHA coating on metallic matrices with their respective theoretical Zn^2+^ concentrations of 5%, 20%, and 0.56 M, observed an increase in ALP activity. The study by Zhong and Ma [55] observed a shift of the enzyme activity peak from the 14th to the 10th day after exposure, compared to the control. On the other hand, Hidalgo-Robatto et al. [29] identified no ALP activity in any of the Zn^2+^ concentrations evaluated, from 2.5% to 10%. The study by Maleki-Ghaleh et al. [38], employing a zinc-containing graphene/nanoHA, also identified strong positive effects in MSC proliferation and release of ALP, but the authors did not provide the ratio of Zn^2+^ incorporation. A similar association of ZnHA with graphene was developed by Chopra et al. [22], and the material osteoinductive property was confirmed by its ability to induce MSCs by in vitro mineralization, and expression of RUNX-2, ALP, BMP-2, Col-1, OCN, and osteopontin (OPN).

Molecular monitoring of RUNX-2, Osterix, OCN, Col-I, and BMP-2 transcription factors reveals more precisely the cell differentiation process and bone formation [70]. These molecules promote the modulation of new tissue construction by maturing the pre-osteoblasts in osteoblasts and matrix producers [68]. Few selected studies have investigated the optimal effect/concentration of Zn^2+^ on osteoblast differentiation pathways and the expression of osteogenic factors. Several studies [22,28,30,37,38,46,53] evaluated osteogenic markers involved in the differentiation of pre-osteoblasts in the presence of osteogenic medium to stimulate differentiation. OCN, a critical protein for calcium deposition in the organic matrix, was increased after exposure to zinc-doped samples (containing 5 to 20% Zn^2+^) [37,46,53], as well as OPN and osteoprotegerin (OPG) [30]. The study by Luo et al. [37], investigating coatings with high concentrations of Zn^2+^, has found an increase in the concentration of OCN, RUNX-2, and Osterix, as well as an increase in the expression of BMP-2 and Col-I after exposure to 20% ZnHA samples. On the other hand, the 30% ZnHA showed a reduction in the concentration of the tested proteins and the genes, most probably due to cytotoxic effects on higher Zn^2+^ doses. The results by Meng et al. [40] showed that Zn^2+^ substitution at 1–2% moderately promoted the MSC differentiation into the osteoblasts, as measured by the ALP/Col-I ratio and expression of OCN, and reduced the osteoclastic activity in co-culture, even with a release of Zn^2+^ at concentrations under 5 ppm. Adipose-derived MSCs also responded to 1% ZnHA on a bilayered hydrogel scaffold by increasing ALP, Col-I, and RUNX-2 expression and in vitro calcium deposition [21].

The deposition of calcium in the organic matrix and the formation of mineralization nodules reveal the complete differentiation of osteoblasts and establish the process of bone formation. The works by Webster et al. [51], He et al. [28], and Wang et al. [49] observed an increase in the deposition of hydroxyapatite by osteoblasts induced by 5% ZnHA, indicating that, at least in vitro, zinc-doped materials affect osteoblast metabolism resulting in increased deposition of mineralization nodules. These results are in accordance with Cuozzo et al. [23], which investigated nanostructured ZnHA/alginate microspheres with theoretical 5% Zn^2+^ content (0.5% final incorporation), which were biocompatible with murine pre-osteoblasts and induced an increase in newly formed bone on a rat calvaria defect in vivo model. Furthermore, these results could help to explain the findings summarized in a systematic review by Cruz et al. [13], where most studies reported that the presence of Zn^2+^ in calcium phosphates improves the production of new bone, even though it depends on the manufacturing process, zinc concentration, and solubility of the materials.

### 4.7. Other Biological Effects

Even though it was not in the scope of the search question of this review, several selected studies have also highlighted the significant antimicrobial effects of ZnHA composites, another trait that renders them promising materials for bone tissue engineering and implant applications in dentistry. Maleki-Ghaleh et al. [38] demonstrated that ZnHA nanoparticles effectively attack bacteria by damaging bacterial membranes, accumulating in the cytoplasm, and increasing reactive oxygen species (ROS) production, with Gram-negative bacteria susceptible. Such an antibacterial effect was also demonstrated for 15% ZnHA nanoparticles [42]. Cuozzo et al. [23] noted that ZnHA composites help prevent post-surgery infections, underscoring their antibacterial properties. Predoi et al. [43] further expanded on these findings by showing that ZnHA, when combined with chitosan and tarragon essential oil, exhibits potent antimicrobial activity against *Escherichia coli*, *Staphylococcus aureus*, and *Candida albicans*, with enhanced effects observed over a 72 h incubation period. Chopra et al. [22] found that incorporating reduced graphene oxide (rGO) with ZnHA significantly reduces bacterial colonies and biofilm formation due to increased ROS production and bacterial cell membrane damage. These studies underscore the potent antimicrobial properties of ZnHA composites, highlighting their potential to improve the safety and efficacy of bone-related medical applications by preventing infections.

The effects of ZnHA on osteoclasts and its relevance to bone regeneration are profound and multifaceted, involving complex interactions with both osteoclasts and osteoblasts. Even though the search strategy of this review focused only on mineralizing cells, some studies presented in vitro evidence of ZnHA influencing osteoclasts significantly, as it initially tends to inhibit osteoclastic activity. For instance, Meng et al. [40] demonstrated that Zn substitution in hydroxyapatite reduced osteoclastic activity in the early stages of co-culture with osteoblasts and osteoclast-like precursor cells. This early inhibition is likely due to the Zn^2+^ interfering with the differentiation pathways of osteoclast precursors, as evidenced by the decreased expression of TRAP5b and IL-1 during the initial phases of co-culture. However, the study also revealed that ZnHA promoted osteoclastic activity at later stages. This paradoxical effect was marked by significantly enhanced expressions of osteoclastic markers such as TRAP5b and IL-1 after prolonged co-culture. The formation of multinucleated osteoclasts was more pronounced in the presence of ZnHA, indicating that zinc plays a role in osteoclasts’ maturation and functional activity over time [40].

The study by Forte et al. [26] investigated the effects of zinc substitution in hydroxyapatite and its multifunctionalization on osteoclasts. It was found that zinc-substituted hydroxyapatite inhibited osteoclast proliferation and activity. The study also highlighted the importance of co-culture systems to mimic the human physiological environment and better understand the interactions between osteoblasts and osteoclasts. Cuozzo et al. [23] evaluated zinc-containing hydroxyapatite composite microspheres and found that zinc reduced bioabsorption rates, which was associated with decreased osteoclastic activity, thereby supporting bone regeneration and repair. The dual role of ZnHA in inhibiting and later stimulating osteoclastic activity is crucial for balanced bone remodeling. Initial inhibition of osteoclasts helps reduce excessive bone resorption immediately after implantation of ZnHA-based materials. Subsequent stimulation of osteoclastic activity ensures that bone resorption and formation are balanced, promoting the remodeling of the new matrix into a more similar structure to natural bone [71]. This dynamic modulation is essential for effective bone regeneration, as it supports the initial phase of new bone formation while preventing long-term deficiencies in bone resorption that could lead to abnormal bone accumulation and poor mechanical properties [72]. In conclusion, ZnHA’s effects on osteoclasts are time-dependent and highly influenced by the presence of osteoblasts, ensuring a balanced bone remodeling process.

### 4.8. Summary of Evidence and Limitations

**Figure 2:**
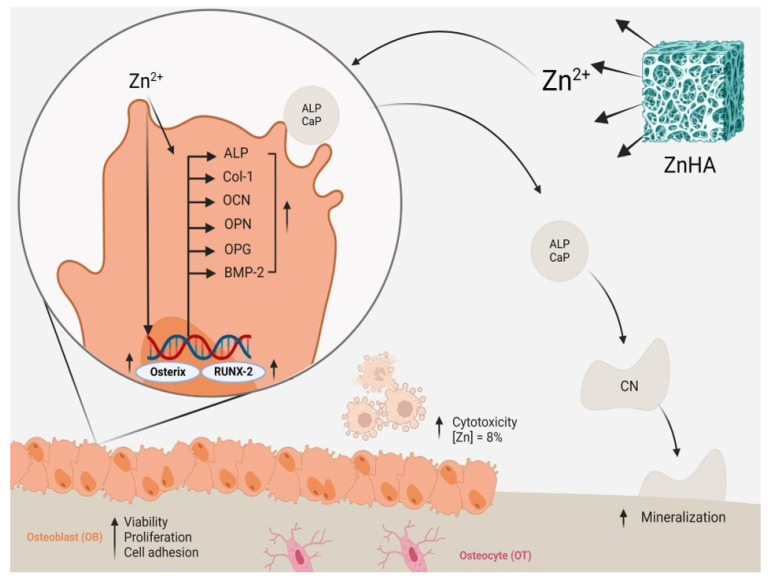
A representative image shows the effects of zinc-doped hydroxyapatite (ZnHA) on osteoblasts and its role in bone regeneration. ZnHA can release Zn^2+^, which directly affects mesenchymal stem cells and osteoblasts, enhancing the expression of RUNX-2, a key transcription factor of osteogenesis. This up-regulation leads to increased levels of bone formation markers such as ALP (Alkaline Phosphatase), Col-1 (Collagen type I), OCN (Osteocalcin), and OP (Osteopontin). The resulting improved osteoblastic activity promotes bone regeneration. However, ZnHA containing zinc above 8% presented cytotoxicity to bone cells.

The literature describes in detail the production processes of synthetic HA by different methods, and the characterization of these materials is thoroughly described, making it possible to understand and reproduce these results. However, biological studies still have significant gaps in the ideal Zn^2+^ concentrations to be incorporated into the materials to stimulate specific biological effects on bone metabolism. The main limitation observed refers to the limited detection of the concentrations released during contact with the biological environment. Only a few studies indeed evaluated the released concentrations of Zn^2+^ in their studies. This gap prevents the complete understanding of the optimal concentrations to enhance the desired biological effects. It may be one of the primary sources of differences and controversies in related pre-clinical in vivo studies. While there is pre-clinical evidence of the positive impact of ZnHA on bone repair [13], the best manufacturing method and ideal Zn^2+^ concentration that can promote bioreaction and osteoconductivity could not yet be assertively determined from the in vitro evidence. Nevertheless, interesting evidence is already available from the identified in vitro studies that allow for tracing the main pathway of effects of zinc-doped HA on osteoblasts and MSCs, as summarized in Figure 2.

Bioresorption of biomaterials is fundamental for the formation of new bone tissue. However, limited data are also available to help understand the impact of biomaterials during osseointegration and biomineralization. Studies show that there are two significant pathways of osteoblast differentiation, WNT/β-catenin and RUNX-2 are both osteogenic activating genes responsible for pre-osteoblast maturation and expression of genes linked to bone differentiation such as BMP-2, Col I, ONC, and OPN [68]. However, there is a lack of evidence on the zinc dose/response of these differentiation pathways in the biological effects of Zn-doped materials. Therefore, further research efforts remain necessary to understand the influence of the level of incorporated zinc during these processes.

This systematic review presents, as its main limitation: (i) a restrictive search key that might have missed some studies related to other complex presentations of HA or ZnHA; (ii) the limitation to in vitro studies, aiming at molecular explanations at the cell level, but that may miss studies with interesting clinical correlations, and (iii) limitation to complete reports, possibly missing potentially interesting results from published abstracts. The search strategy avoided the inclusion of in vivo studies, which could greatly contribute to detecting the impacts of the osteoblast response to ZnHA on new bone formation, material resorption, inflammatory responses, as well as other effects. Nevertheless, the review by Cruz et al. [13] presents a comprehensive discussion of animal studies on the effects of ZnHA. Regardless of these limitations, this search strategy allowed us to conclude that incorporating zinc into hydroxyapatite significantly enhances the cellular response, promoting bone regeneration and osteointegration behaviors. This systematic review has identified in vitro evidence that ZnHA stimulates osteoblast proliferation, adhesion, and differentiation, which are critical for effective bone healing. Studies consistently show that ZnHA exhibits excellent biocompatibility despite presenting cytotoxicity at theoretical incorporations above 8% of zinc. This association stimulates ALP activity and the expression of bone growth factors in osteoblasts and MSCs on ranges from 5 to 20% of zinc theoretical incorporation. However, the notable variability in the concentrations of zinc used across different studies complicates the identification of an ideal doping level for clinical applications. Future research should focus on evaluating the zinc incorporation and release level to ensure consistent and effective outcomes in bone regeneration. Zinc-doped hydroxyapatite materials are promising candidates for bone repair and regeneration applications. The evidence supports their potential to improve osteoblast function and bone tissue integration, though further studies are necessary to optimize their formulation for safe and efficient clinical use.

## Figures and Tables

**Table 1:** Relationship between eligibility criteria and the total number of excluded entries.

Eligibility Criteria	Number of Excluded Entries (N)
Reviews articles, book chapters, and theses	102
Without zinc or a mixture of ions	121
Only physical-chemical tests	43
Only in vivo	19
Bacteriological	12
Other materials	169
Off-topic	103
Only abstract	4

**Table 2:** Quality assessment of the selected studies according to the Toxicological Data Reliability Advisory Tool (ToxRTool).

Reference	Group I: Test Substance Identification [4]	Group II: Test System Characterization [3]	Group III: Study Design Description [7]	Group IV: Study Results Documentation [3]	Group V: Plausibility of Study Design and Data [2]	Total Score	Reliability Categorization
Bakhsheshi-Rad et al. [17]	3	2	3	2	1	11	Reliable with restrictions
Begam et al. [18]	4	3	5	3	2	17	Reliable
Bhattacharjee et al. [19]	4	3	6	3	2	18	Reliable
Bhowmick et al. [20]	4	3	6	2	1	16	Reliable
Cao et al. [21]	4	3	5	3	2	17	Reliable
Chopra et al. [22]	4	2	6	3	2	17	Reliable
Cuozzo et al. [23]	4	2	5	3	2	16	Reliable
Ding et al. [24]	2	3	5	3	1	14	Reliable with restrictions
Dittler et al. [25]	4	2	5	3	2	16	Reliable
Forte et al. [26]	4	3	6	3	2	18	Reliable
Ghorbani et al. [27]	4	2	4	3	1	14	Reliable with restrictions
He et al. [28]	4	3	6	3	2	18	Reliable
Hidalgo-Robatto et al. [29]	4	3	5	3	1	16	Reliable
Hou et al. [30]	2	2	6	3	2	15	Reliable
Huang et al. [31]	4	3	6	3	2	18	Reliable
Kazimierczak et al. [32]	4	3	6	3	2	18	Reliable
Li et al. [33]	4	2	5	3	1	15	Reliable
Li et al. [34]	3	3	5	3	1	15	Reliable
Lima et al. [35]	2	2	6	3	2	15	Reliable
Liu et al. [36]	4	1	6	3	2	16	Reliable
Luo et al. [37]	1	2	3	3	2	11	Reliable with restrictions
Maleki-Ghaleh et al. [38]	4	2	6	3	1	16	Reliable
Mavropoulos et al. [39]	3	3	4	3	2	15	Reliable
Meng et al. [40]	4	3	6	3	2	18	Reliable
O’Sullivan et al. [41]	4	2	4	3	1	14	Reliable with restrictions
Okada et al. [42]	4	3	6	3	2	18	Reliable
Predoi et al. [43]	4	3	5	3	2	17	Reliable
Santos et al. [44]	3	3	5	3	1	15	Reliable
Santos et al. [45]	4	2	4	3	2	15	Reliable
Thian et al. [46]	3	3	5	3	2	16	Reliable
Ullah et al. [47]	4	2	6	3	2	17	Reliable
Valarmathi and Sumathi [48]	4	2	6	3	2	17	Reliable
Wang et al. [49]	4	3	5	3	2	17	Reliable
Wang et al. [50]	4	2	5	3	2	16	Reliable
Webster et al. [51]	3	3	5	3	2	16	Reliable
Webster et al. [52]	4	3	6	3	2	18	Reliable
Yang et al. [53]	2	2	5	3	1	13	Reliable with restrictions
Zhang [54]	3	3	5	2	1	14	Reliable with restrictions
Zhong and Ma [55]	3	2	5	3	2	15	Reliable

**Table 3:** Characteristics of biomaterials and chemical compounds in the selected studies.

Reference	Material	Theoretical/Real Amount of Zn^2+^ Added to the Biomaterial	Zn^2+^ Incorporation Method	Amount of Zn^2+^ Released into Media
Bakhsheshi-Rad et al. [17]	Coating of titanium nanoparticle	Not indicated	Immersion in a solution of 100 ppm of ZnCl_2_	57 ppm
Begam et al. [18]	Blocks	5%/2.9%	Precipitation of 5 weight % ZnO	Not indicated
Bhattacharjee et al. [19]	Coating of titanium disc	0.10, 0.25, and 0.5 wt%/Not indicated	Mechanochemical synthesis	Not indicated
Bhowmick et al. [20]	Nanocomposite/CTS-PEG-HAP-ZnO ^1^	5%, 10%, and 15%/Not indicated	Nanoparticles of ZnO were prepared by Zn(OAc)_2_ (0.45 M)	Not indicated
Cao et al. [21]	Bilayer hydrogel scaffold	ZnHA ^2^–0.1%, 0.5%, and 1.0% (*w*/*v*)/Not indicated	Precipitation of Zn(NO_3_)_2_	Not indicated
Chopra et al. [22]	Nanoparticle	ZnHA 2.76%/Not indicated	Hydrothermal synthesis	Not indicated
Cuozzo et al. [23]	Microspheres	ZnHA 0.5%/Not indicated	Precipitation of Zn(NO_3_)_2_	Not indicated
Ding et al. [24]	Coating of titanium discs	ZnHA–Not indicated/1.33 wt%	Precipitation of 0.05 M Zn(NO_3_)_2_	Not indicated
Dittler et al. [25]	Coating of bioglass	ZnHA–Not indicated/10,800 ppm	Precipitation of Zn(NO_3_)_2_	Not indicated
Forte et al. [26]	Disc	Not indicated	Precipitation 1.08 M of Zn(NO_3_)_2_	Not indicated
Ghorbani et al. [27]	Scaffold/PCL-Ch-nZnHA ^3^	5%/Not indicated	Precipitation of Zn(NO_3_)_2_	Not indicated
He et al. [28]	Film/Titanium	Not indicated	Precipitation of 0.1 mol/L Zn(NO_3_)_2_	F-ZCP ^4^ 0.2 μg/mL - TiO_2_/D-ZCP ^5^ 0.10 μg/mL -TiO_2_/S-ZCP ^6^-0.08 μg/ml
Hidalgo-Robatto et al. [29]	Coating of titanium disc	ZnHA 2.5%/0.15% ZnHA 5.0%/0.99% ZnHA 7.5%/0.88% ZnHA 10%/1.89%	Mixture of ZnO with commercial HA	Not indicated
Hou et al. [30]	Coating of titanium implants	ZnHA–2.5%/release	Precipitation of Zn(NO_3_)_2_	Not indicated
Huang et al. [31]	Powder	15%/9.21	Precipitation of Zn(NO_3_)_2_	120 mg/L
Kazimierczak et al. [32]	Chitosan-agarose-doped HA scaffold	ZnHA 1 mol/Not indicated	Precipitation of Zn(NO_3_)_2_	Chit/Aga/HA-Zn–4.42 μg/mL
Li et al. [33]	Glass powders	CaO-P_2_O_5_-ZnO-Na_2_O 2 mol%/Not indicated	Precipitation of 2 mol% Zn(NO_3_)_2_	3.58–3.54 ppm
Li et al. [34]	Powder	ZnHA (1%, 2%, 4%, and 8%)	Precipitation different values of Zn(NO_3_)_2_ 2 M	Not indicated
Lima et al. [35]	Granules	ZnHA 1%/0.1%	Precipitation of Zn(NO_3_)_2_	1 ppm
Liu et al. [36]	Coating of titanium discs	Not indicated	VIPF-APS ^7^ Technique	Not indicated
Luo et al. [37]	Coating of titanium discs (Tixos–commercial titanium)	ZnHA 10%/Not indicated ZnHA 20%/Not indicated ZnHA 30%/Not indicated	Precipitation of Zn(NO_3_)_2_	Not indicated
Maleki-Ghaleh et al. [38]	Nanoparticles	Not indicated	Mechanochemical	Not indicated
Mavropoulos et al. [39]	Disc	ZnHA–Not indicated/2.3%	Precipitation of Zn(NO_3_)_2_	Not indicated
Meng et al. [40]	Coating of titanium discs	1% and 2%/Not indicated	Precipitation of Zn(NO_3_)_2_	1% ZnHA/TiO–3.8 ppm, 2% ZnHA/TiO–5.5 ppm
O’Sullivan et al. [41]	Coating of titanium discs	Not indicated/9 ppm	Deposition of ZnHA powder	0.45 ppm
Okada et al. [42]	Powder	ZnHA 5%/4.3% ZnHA 10%/9.2% ZnHA 15%/14.7%	Precipitation of Zn(NO_3_)_2_	Not indicated
Predoi et al. [43]	Powder	Not indicated/0.06%	Precipitation of Zn(NO_3_)_2_	Not indicated
Santos et al. [44]	Composite (Nanoparticle and Collagen)	Not indicated	Precipitation of 0.158 mol% Zn(NO_3_)_2_	Not indicated
Santos et al. [45]	Composite (Nanoparticle and Collagen)	Not indicated	Precipitation of 1.0 mol% Zn(NO_3_)_2_	Not indicated
Thian et al. [46]	Disc	ZnHA 1.5%/1.6%	Precipitation of Zn(NO_3_)_2_	Not indicated
Ullah et al. [47]	Disc	ZnHA 5%/2.66%	Precipitation of Zn(NO_3_)_2_	ZnHA_1_ 5%–1 mg/L
Valarmathi and Sumathi [48]	Nanocomposite/Zn-HAP/SF/MC^8^	(Ca + Zn) 1 mM	Precipitation of Zn(NO_3_)_2_	Not indicated
Wang et al. [49]	Nanoparticle	PAA-CaP/Zn^9^ 5%/Real concentration–Not indicated	Precipitation of ZnCL_2_ (0.0000061 M)	Not indicated
Wang et al. [50]	Rods/Nanoparticles	Z1-0.0056 mM/1.2 × 10^−4^ μg/cm^2^ Z2-0.056 mM/0.06 μg/cm^2^ Z3-0.56 mM/0.195 μg/cm^2^	Precipitation of ZnCL_2_ (0.0056 mM, 0.056 mM, 0.56 mM)	Not indicated
Webster et al. [51]	Disc	5%/0.7%	Precipitation of Zn(NO_3_)_2_	Not indicated
Webster et al. [52]	Borosilicate glass coverslips/covered ZnHA	2%/Not indicated	Precipitation (Concentration and solution source of Zn not indicated)	Not indicated
Yang et al. [53]	Plates	10%/1.04%	Precipitation of 10% Zn(NO_3_)_2_	Not indicated
Zhang [54]	Coating of titanium discs	ZnHA/TiO^−^ (0.0025 M), ZnHA/TiO^2−^ (0.005 M), and ZnHA/TiO^3−^ (0.01 M)/Not indicated	Dipping the films in 0.0025 M, 0.005 M, and 0.01 M Zn(NO_3_)_2_	ZnHA/TiO^−^ 0.058 ZnHA/TiO^2−^ 0.06, and ZnHA/TiO^3−^ 0.066
Zhong and Ma [55]	Nanoparticle coating of Titanium sheets	ZnHA 2%/2.0% ZnHA 5%/2.4%	Precipitation of 0.1 mol/L Zn(NO_3_)_2_	Not indicated

^1^ CTS-PEG-HAP-ZnO = chitosan, poly(ethylene glycol), and nano-hydroxyapatite–zinc oxide; ^2^ ZnHA = zinc-doped hydroxyapatite; ^3^ PCL-Ch-nZnHA = chitosan, poly(ɛ-caprolactone), and zinc-doped hydroxyapatite nanoparticles; ^4^ F-ZCP = fully covered Zn-CaP; ^5^ D-ZCP = densely distributed Zn-CaP; ^6^ S-ZCP = sparsely distributed Zn-CaP; ^7^ VIPF-APS = vapor-induced pore-forming atmospheric plasma spraying; ^8^ Zn-HAP/SF/MC = silk fiber, methylcellulose, and zinc substituted hydroxyapatite; ^9^ PAA-CaP/Zn = Poly(acrylic acid) modified Zn-doped calcium phosphate nanoparticles.

**Table 4:** Physicochemical characterization of zinc incorporation in the selected studies.

Reference	Elemental Analysis	Composition	Crystallinity	Degradation/ Dissolution	Outcome
Bakhsheshi-Rad et al. [17]	EDX ^1^	-	XRD ^2^	ICP-OES ^3^/SBF ^4^	EDX—incorporation of Zn in ZnHA, ratio (Zn + Ca)/P = 1.54; XRD—characteristic parameters of HA; there was a reduction of the ZnHA crystallite. SBF—the samples suffered corrosion, releasing Zn (57 ppm)
Begam et al. [18]	AAS ^5^	FTIR ^6^	XRD	-	AAS—detected the presence of Zn in the HA; XRD—crystallinity reduced after incorporation of Zn; FTIR—intensity of the phosphate group decreased after Zn incorporation
Bhattacharjee et al. [19]	-	-	XRD	-	XRD—confirms the conditions for the formation and retention of ZnHA characteristics
Bhowmick et al. [20]	-	FTIR	XRD	-	FTIR—identified the presence of ZnO NPs associated with HA; XRD—confirmed the presence of peaks corresponding to ZnO. Thus, there was no change in the crystalline network of the HA
Cao et al. [21]	EDX	-	XRD	-	XRD—characteristic Ca/P peaks and reduction in intensity indicate the incorporation of Zn; EDX—identified the presence of Zn in the sample
Chopra et al. [22]	EDX	FTIR	XRD	ICP-MS ^7^	XRD—characteristic Ca/P peaks. Changes in the XRD peaks, in the morphology of the FESEM ^8^ crystals, and the Raman bands indicate the replacement of Ca by Zn in the HA structure; ICP—revealed the release of Zn within 60 days
Cuozzo et al. [23]	EDX	-	XRD	-	XRD—characteristic Ca/P peaks; EDX-identified peaks characteristic of the presence of Zn
Ding et al. [24]	EDX	FTIR	XRD	-	EDX —the Zn/P molar ratio of 0.053; XRD—Incorporation of Zn reduction of cell HA parameters. Peaks correspondents of HA; FTIR—HA characteristic bands, presence carbonate group, the sample was calcium deficient
Dittler et al. [25]	ICP-AES ^9^/XPS ^10^	FTIR	XRD	ICP-AES/PBS ^11^	TEM ^12^—confirmed the crystalline formation of the material; EDX and XPS—confirmed the presence of Zn in the material. Changes in the Ca/P and Ca/O ratio indicated the incorporation of Zn into the material; FTIR—characteristic Ca/P bands; ICP-AES—quantified the incorporation of Zn into Ca/P and the release of low doses of Zn in PBS solution
Forte et al. [26]	ICP-AES	Raman	XRD	-	XRD—characteristic Ca/P peaks without changes after the addition of PEI, without changes in the crystalline structure; ICP-AES—proved the presence of Zn^2+^ in the material structure; Raman—presented corresponding Ca/P groups
Ghorbani et al. [27]	FESEM/EDX	ATR-FTIR ^13^	XRD	-	XRD—presence of specific hydroxyapatite peaks; ATR-FTIR—confirmed the presence of calcium phosphate in the nanocomposite; EDX—confirmed the presence of Zn in the material
He et al. [28]	-	-	XRD	ICP-MS/Tris-HCL	XRD—suggests low crystallinity of Zn-HA; ICP-MS—gradual release of Zn^2+^
Hidalgo-Robatto et al. [29]	EDX	FTIR	XRD	-	EDX—reveals the presence of zinc; XPS_9_—quantified a Zn/Ca molecular ratio between 0.01 and 0.11; XRD—parameter characteristic of HA and Zn-doped HA decreases the crystal size; FTIR—spectra corresponded to HA
Hou et al. [30]	-	-	-	-	No description of the physical-chemical characterization
Huang et al. [31]	EDX/XRF ^14^/XPS	FTIR	XRD	ICP-AES/PBS	XRD and ATR-FTIR—confirmed characteristic Ca/P peaks and bands, and metal substitution did not interfere with the formation of nanocrystals; EDX—uniform distribution of Zn on the surface of the material; ICP-AES—confirms the gradual release of zinc at low doses
Kazimierczak et al. [32]	-	FTIR/PXRD	PXRD ^15^	Spectrophotometer/alpha-MEM	PXRD—characteristic Ca/P peaks and presence of only one crystalline phase. Addition of Zn did not change the crystallinity of the material; FTIR—presence of characteristic Ca/P bands and reduction in the intensity of the OH- band suggests the replacement of Zn in the HA structure; ICP-AES—identified the presence of Zn after the production of scaffolds
Li et al. [33]	EDX	-	XRD	AAS/SBF	XRD—shows peak characteristics of HA, amorphous structure; AAS—gradual release of Zn in SBF solution. EDX- analyzed the new apatite formation; (Zn + Ca)/P = 1.55, sample deficient in Ca
Li et al. [34]	EDX	FTIR	XRD	-	EDX—revealed the presence of zinc and suggested incorporation in HA structure; XRD—Different concentrations of doped zinc represent a variation in crystallinity parameters; FTIR—zinc doping did not modify the internal structure
Lima et al. [35]	AAS	FTIR	XRD	AAS/DMEM ^16^	AAS—revealed the incorporation of 0.1% of the Zn in HA; XRD—typical crystallinity of HA. No indicated Zn doped and possible modification; FTIR—indicated monophasic sample; AAS—after 24 h, 0.8 ppm of Zn was released in solution
Liu et al. [36]	FESEM/EDX	FTIR	XRD	-	XRD—characteristic Ca/P peaks; FTIR—identified the presence of characteristic Ca/P bands; EDX—identified the presence of Zn in the sample
Luo et al. [37]	-	-	SEM ^17^	-	Crystal lattice contraction
Maleki-Ghaleh et al. [38]	XPS	ATR-FTIR	XRD	-	XRD and ATR-FTIR—confirmed characteristic Ca/P peaks and bands
Mavropoulos et al. [39]	XRF	ATR-FTIR	XRD	-	XRF—Zn-HA presented Zn in 2.3%; XRD—parameter compatible with HA. Zn-doped reduced the crystallite size; ATR-FTIR—shows HA characteristic groups and detects the adsorbed proteins
Meng et al. [40]	-	-	XRD	AAS/DMEM	XRD—presence of peaks related to Ca/P phosphate, indicative of zinc replacement in the material’s structure; AAS/DMEM—low doses of Zn were identified in the culture medium for cell exposure. In 28 days, the Zn concentration was reduced in the supernatant
O’Sullivan et al. [41]	EDX, XPS, and ICP-OES	-	-	ICP-OES/PBS	XPS, EDX, and ICP-OES—different quantified concentrations of zinc doped into HA. The variation corresponds to the different techniques; ICP-OES—Zn-HA released only 10% of the Zn doped into HA
Okada et al. [42]	ICP-AES	FTIR	XRD	-	XRD—indicated the presence of characteristic Ca/P peaks. Changes in peak intensity were related to incorporating Zn into the HA structure. The increase in Zn concentration altered the crystalline formation of the material; FTIR—identified characteristic Ca/P bands. Changes in the vibration of the OH- bands showed shortening with increasing Zn incorporation; ICP-AES—confirmed the presence of Zn in the samples with an incorporation efficiency of 85% relative to the expected concentration of metal incorporation
Predoi et al. [43]	-	FTIR	XRD	-	XRD—suggests low crystallinity and characteristic peaks; FTIR—showed specific bands of calcium phosphate; EDX—presence of zinc in samples
Santos et al. [44]	-	-	-	-	No description of physical-chemical characterization
Santos et al. [45]	-	-	-	-	No description of physical-chemical characterization
Thian et al. [46]	XRF, XPS	FTIR	SAED ^18^, XRD	-	SAED—polycrystalline-shaped crystals. Substitution of Zn in HA has been shown to reduce the size of HA crystals. There was no formation of new crystalline phases; FTIR—shows corresponding compounds of HA; XRF and XPS—show the incorporation of Zn; XRF—quantified Zn in 1.6 wt%
Ullah et al. [47]	ICP-AES	ATR-FTIR	XRD	ICP-AES/PBS	XRD—indicated the presence of characteristic Ca/P peaks, with changes in intensity following the incorporation of Zn. Latitude parameters (a) and (c) indicate the presence of Zn in the material structure and reduction in crystallinity; FTIR—showed characteristic Ha peaks after Zn incorporation; ICP-AES—revealed the incorporation and release of Zn from the material
Valarmathi and Sumathi [48]	EDX	FTIR	XRD	-	XRD—characteristic Ca/P peaks and changes in peak intensities reveal the variation in Zn incorporation into the material structure. Identified the reduction in crystallite size from peak analysis; FTIR—identified the characteristic Ca/P groups
Wang et al. [49]	ICP-AES and EDX	FTIR	XRD	-	XRD—crystalline structure evaluation, while not quantified. Characteristic peaks were similar to HA; FTIR—analysis of functional groups; EDX—confirmed the presence of Zn; ICP-AES—quantified Zn/Ca atomic ratio at 5.85%
Wang et al. [50]	XPS and ICP-AES	-	XRD	-	XPS confirmed the presence of Zn; ICP-AES quantified Zn. The addition of Zn decreases the crystal size.
Webster et al. [51]	EDX	-	XRD	-	EDX—incorporation of 0.7% form Zn; XRD—crystal reduction after incorporation of Zn
Webster et al. [52]	-	-	-	-	No indication of any results that prove Zn incorporation or structural changes
Yang et al. [53]	ICP-AES	-	-	-	ICP-AES—indicated the ratio of Zn/(Ca + Zn), molar ratio 1.04%
Zhang [54]	-	-	XRD	Plasma spectrometer/Tris-buffer solution (0.05 M)	With the increase of Zn incorporation, the HA phase was reduced. Zinc can be gradually released
Zhong and Ma [55]	EDX	FTIR	TEM, XRD	-	The presence of Zn in the SF-2% ZnHA and SF-5% ZnHA samples was 2% and 2.4%, respectively. The crystallinity parameters were not detailed. With the addition of Zn, Zn-HA agglomerates increased slightly after exposure to SBF, and bone-like apatite formed.

^1^ EDX = energy dispersive X-ray spectroscopy; ^2^ XRD = X-ray diffractometry; ^3^ ICP-OES = inductively coupled plasma optical emission spectroscopy; ^4^ SBF = simulated body fluid; ^5^ AAS = atomic absorption spectroscopy; ^6^ FTIR = Fourier transform infrared spectroscopy; ^7^ ICP-MS = inductively coupled plasma mass spectrometry; ^8^ FESEM = field emission scanning electron microscopy; ^9^ ICP–AES = inductively coupled plasma atomic emission spectroscopy; ^10^ XPS = X-ray photoelectron spectroscopy; ^11^ PBS = Phosphate buffer-saline; ^12^ TEM = transmission electron microscope; ^13^ ATR-FTIR = Fourier transform infrared spectroscopy attenuated total reflectance microscopy; ^14^ XRF = X-ray fluorescence; ^15^ PXRD = powder X-ray diffraction; ^16^ DMEM = Dulbecco’s modified eagle medium; ^17^ SEM = scanning electron microscopy; ^18^ SAED = selected area electron diffraction.

**Table 5:** In vitro evaluation of the biological effects of zinc incorporation in the selected studies.

Reference	Cell Type	Biological in Vitro Tests	Genes/Proteins Assessed	Outcome
Bakhsheshi-Rad et al. [17]	MG-63 (human osteosarcoma cell line)	MTT ^1^ and FM ^2^ (DAPI ^3^)	-	Cells showed high affinity and cytocompatibility
Begam et al. [18]	MG-63	Alamar Blue, MTT, CLSM ^4^, SEM	-	Increased viability and adhesion on ZnHA 1250; increased cell growth compared to the control
Bhattacharjee et al. [19]	hFOB (human fetal osteoblast cells)	MTT and FESEM	-	No significant variation in viability compared to the control. Cells presented a healthy morphology due to the presence of phyllodes
Bhowmick et al. [20]	Erythrocytes and MG-63	Hemolytic assay, MTT	-	Cytocompatibility with blood; increased cell viability; no significant increase in proliferation
Cao et al. [21]	hADMSCs (human adipose mesenchymal stem cells)	FM (Live/dead), CCK-8 (WST-8) ^5^	ALP ^6^, OPN ^7^, and RUNX2 ^8^	High biocompatibility, proliferation, and cell adhesion on the scaffold with ZnHA; Greater mineralization and expression of osteogenic markers in the SBH2 material (0.5% ZnHA)
Chopra et al. [22]	L929 (mouse fibroblast cell line); MSCs (mesenchymal stem cells); HUVECs (Human umbilical vein endothelial cells)	MTT, FM, and SEM	MSCs: ALP, COL1 ^9^, BMP2 ^10^, OCN ^11^, OPN, and mineralization; HUVECs: cell migration, capillary formation, and gene expression (FGF2 ^12^, VEGFA ^13^, and PDGF ^14^)	High biocompatibility with G_3_HapZn in all cell types; MSCs—increased expression of genes associated with bone formation: ALP (7 d); COL1 (7 and 14 d); RUNX2 (14 d and 21 d); BMP2 (14 d); OCN (7 d, 14 d and 21 d). Increased mineralization at 7 d and 14 d; HUVECs—increased angiogenesis, cell migration, and gene expression: FGF2, VEGFA, and PDGF in the presence of G_3_HapZn
Cuozzo et al. [23]	MC3T3-E1 (mouse preosteoblasts cell line)	PrestoBlue	-	No significant difference in cell viability
Ding et al. [24]	MC3T3-E1	MTT and SEM	-	Typical cell morphology, with filipodia to anchor cells/increase cell viability
Dittler et al. [25]	MG-63	WST8, BrdU ^15^, and SEM	-	No significant variation in cell viability compared to the control and low rate of cell adhesion; the association of metals Mg and Zn (Mg-Zn-HA-BG) showed a significant increase in all parameters, indicating synergy between the metals
Forte et al. [26]	MG-63 and 2T-110 (human osteoclast precursor cell line)	WST-1 ^16^, MF	ALP, COL1, OPG ^17^, RANKL ^18^, TRAP ^19^	ZnHa showed low cell recovery capacity after exposure to H_2_O_2_; ZnHA did not present significant variation in the quantification of the biomarkers evaluated
Ghorbani et al. [27]	hADMSCs	SEM and MTT	-	Cell adhesion; Zn concentration was not toxic; proliferation decrease
He et al. [28]	MC3T3-E1	Alamar Blue and SEM	ALP, OCN, Col-1, Runx-2	Morphology showed filopodia; increased viability; increased APL activity, and OCN secretion/TiO2/S-ZCP showed better performance in all evaluated cytokines
Hidalgo-Robatto et al. [29]	MC3T3-E1	MTT, SEM, and CLSM (F-actin)	ALP	Adhesion and proliferation similar to control, good viability, and similar ALP activity between samples
Hou et al. [30]	HEPM (human embryonic palatal mesenchymal)	-	COL1A1 (COL1), TNFRSF11B (OPG), SPP1 (OPN)	Significant increase in the expression of COL1A1 (COL1) at 3 d and TNFRSF11B (OPG) at 7 d and 11 d
Huang et al. [31]	MSCs, OBs (osteoblasts), MC3T3-E1, 143b, MG-63, and UMR-106 (mouse osteosarcoma epithelial-like cell line)	Alamar Blue, CCK-8, FM	ALP, COL1, OCN, RUNX2, OPN	Extract biocompatibility and no changes in cell proliferation (5 d reduction of proliferation); high cell adhesion and spreading; low ALP activity compared to the control, but greater deposition of Ca nodules; osteogenic factors with lower expression compared to the Se/Sr/Zn-HA material
Kazimierczak et al. [32]	MC3T3-E1, BMDSCs (bone marrow-derived stem cells), and hADSCs	MTT, CLSM	COL1, ALP, and OCN	Chit/aga/HA-Zn—showed biocompatibility high cell spread with the extract (Zn 4.42 μg/mL); in contact with the scaffold, it showed low cell adhesion; no significant increase in COL1, ALP, and OCN rates
Li et al. [33]	MC3T3-E1	SEM	-	Morphology typical, with filopodia and lamellipodia; increased proliferation, compared to the control, but decreased compared to the other samples
Li et al. [34]	MC3T3-E1	CCK-8	-	Decreased cell viability with high Zn concentration, but increased proliferation in Zn-HA 1%; morphology similar to the control in Zn-HA 1%
Lima et al. [35]	Balb/3T3 Clone A3 (mouse embryonic fibroblasts), primary human OBs, and human monocytes	XTT ^20^, NR ^21^, CVDE ^22^, apoptosis assay, and SEM	-	High cell viability and integrity membrane; no significant apoptosis; no difference in cell adhesion
Liu et al. [36]	hFOBs	MTT	ALP	More significant proliferation in the Zn-Hap-Ti group at 3 d. The group with the association of Zn, Sr, and Mg ions (ZnSrMg-Hap-Ti) showed more significant proliferation and ALP activity
Luo et al. [37]	MG-63	FM (DAPI), CCK-8 (WST-8) and Total protein quantification	ALP, RUNX2, Osterix, OCN, COL1, and BMP2	Increased proliferation, cell adhesion, and total protein quantification in ZnHA 20%. Osteogenic factors were increased by ZnHA 20%.
Maleki-Ghaleh et al. [38]	MSCs	MTT	ALP	Significant increase in cell proliferation and ALP activity in the presence of Zn-doped samples
Mavropoulos et al. [39]	MC3T3-E1	XTT, NR, CVDE, SEM, FM	-	No significant difference in cell viability; morphology similar to the control and high adhesion; increased cell spreading and actin fibers formation
Meng et al. [40]	BALB/cBMSC (BMSCs isolated from healthy BALB/c mice cell line, osteoblasts) and Raw264.7 (murine macrophage from blood, osteoclasts precursors)	FM, MTT, LDH ^23^	ALP, COL1, TRAP5, OCN, IL-1 ^24^, TNF-α ^25^, PTH ^26^, RANKL	Osteoblasts—increase in viability, proliferation, and expression of ALP/COL1 and OCN in the presence of 1% and 2% ZnHA. Osteoclasts—significant increase in RANKL and TRAP5b at 21 d (co-culture), with stabilization at 28 d. Lower expression of IL-1 and TNF-α with no significant variation
O’Sullivan et al. [41]	MG-63	MTT	-	Cytocompatibility and increase in proliferation
Okada et al. [42]	MC3T3-E1	WST-8	-	Significant increase in cell proliferation after exposure to 0.1 mg/mL of Zn(15)-Hap
Predoi et al. [43]	hFOB 1.19	FM and MTT	-	High biocompatibility; Morphology of the cells showed no changes in their structure, and the presence of lamellipodia and filopodia was observed
Santos et al. [44]	MC3T3-E1	MTT	ALP	No significant difference compared to the control
Santos et al. [45]	hOB	MTT	-	Cytocompatibility is similar to the control
Thian et al. [46]	ADMSCs	Alamar Blue, CLSM, SEM	COL1, OCN	Increased cell viability/similar to the control/showed evidence of biomineralization similar to the control/OCN expression increase in ZnHA
Ullah et al. [47]	MC3T3-E1	CCK-8, FM, SEM	-	Less proliferation and adhesion were observed compared to the control group, with increased ALP activity. When co-substituted with Mg, it showed an increase in all analyzed parameters, indicating synergy between the metals
Valarmathi and Sumathi [48]	MG-63	MTT	-	High biocompatibility of the optimized material (SM10 [Zn1.0]) at a concentration of 25 μg/mL. However, the concentration did not show significant variation compared to the control
Wang et al. [49]	rADSC (rat adipose-derived stem cells)	Alamar Blue, SEM, ARS ^27^	ALP	Cell viability similar to the control; favorable to adhesion and proliferation; increased mineral deposition, significantly after ten days
Wang et al. [50]	NIH3T3 and MC3T3-E1	CCK-8 (WST-8), MF, Bradford test	ALP	Fibroblasts: cell density increase; osteoblasts: increased DNA content and total proteins; cellular response was higher with the higher concentrations of Zn and increased significantly after three weeks
Webster et al. [51]	Human Osteoblasts (hOBs)	FM, BCA ^28^ Assay, Calcium assay	ALP	No difference in adhesion; increased mineral deposition; decreased APL expression as compared to all samples
Webster et al. [52]	MC3T3-E1	Coomassie Brilliant Blue	-	Increased cell adhesion
Yang et al. [53]	MC3T3-E1	Total protein quantification	ALP and OCN	Increased cell proliferation; increased ALP activity and OCN production (14 d)
Zhang [54]	MG-63	MTT, SEM, and MF	-	increase cellular proliferation; characteristic morphology of differentiated cells with filopodia and grasped the surface; better adhesion
Zhong and Ma [55]	MC3T3-E1	CCK-8 (WST-8), MF and SEM	ALP	Increased cell proliferation and ALP activity; ZnHA facilitated adhesion and proliferation, but adhesion is similar in all samples

^1^ MTT = 3-(4,5-Dimethylthiazol-2-yl)-2,5-Diphenyltetrazolium Bromide; ^2^ FM = fluorescence microscopy; ^3^ DAPI = 4′,6-diamidino-2-phenylindole; ^4^ CLSM = confocal laser scanning microscope; ^5^ CCK-8 (WST-8) = cell proliferation and cell toxicity (WST-8); ^6^ ALP = alkaline phosphatase; ^7^ OPN = osteopontin; ^8^ RUNX2 = runt-related transcription factor 2; ^9^ COL1 = collagen type 1; ^10^ BMP2 = bone morphogenic protein 2; ^11^ OCN = osteocalcin; ^12^ FGF2 = fibroblast growth factor 2; ^13^ VEGFA = vascular endothelial growth factor A; ^14^ PDGF = platelet-derived growth factor; ^15^ BrdU = bromodeoxyuridine; ^16^ WST-1 = cell proliferation reagent (WST-1); ^17^ OPG = osteoprotegerin; ^18^ RANKL = receptor activator of nuclear factor kappa beta (NFkB ligand); ^19^ TRAP = tartrate-resistant acid phosphatase; ^20^ XTT = 2,3-Bis-(2-Methoxy-4-Nitro-5-Sulfophenyl)-2H-Tetrazolium-5-Carboxanilide; ^21^ NR = neutral red; ^22^ CVDE = crystal violet dye elution; ^23^ LDH = lactate dehydrogenase; ^24^ IL-1 = interleukin-1; ^25^ TNFα = tumour necrosis factor α; ^26^ PTH = parathyroid hormone; ^27^ ARS = alizarin red staining; ^28^ BCA = bicinchoninic acid.

## Data Availability

The data are within the article and available to the corresponding authors upon request.

## Supplementary Materials

The following supporting information can be downloaded at: https://www.mdpi.com/article/10.3390/jfb15070178/s1.
